# A Multi-Sample Based Method for Identifying Common CNVs in Normal Human Genomic Structure Using High-Resolution aCGH Data

**DOI:** 10.1371/journal.pone.0026975

**Published:** 2011-10-31

**Authors:** Chihyun Park, Jaegyoon Ahn, Youngmi Yoon, Sanghyun Park

**Affiliations:** 1 Department of Computer Science, Yonsei University, Seoul, South Korea; 2 Division of Information Engineering, Gachon University of Medicine and Science, Incheon, South Korea; Université Paris-Sud, France

## Abstract

**Background:**

It is difficult to identify copy number variations (CNV) in normal human genomic data due to noise and non-linear relationships between different genomic regions and signal intensity. A high-resolution array comparative genomic hybridization (aCGH) containing 42 million probes, which is very large compared to previous arrays, was recently published. Most existing CNV detection algorithms do not work well because of noise associated with the large amount of input data and because most of the current methods were not designed to analyze normal human samples. Normal human genome analysis often requires a joint approach across multiple samples. However, the majority of existing methods can only identify CNVs from a single sample.

**Methodology and Principal Findings:**

We developed a multi-sample-based genomic variations detector (MGVD) that uses segmentation to identify common breakpoints across multiple samples and a *k-means*-based clustering strategy. Unlike previous methods, MGVD simultaneously considers multiple samples with different genomic intensities and identifies CNVs and CNV zones (CNVZs); CNVZ is a more precise measure of the location of a genomic variant than the CNV region (CNVR).

**Conclusions and Significance:**

We designed a specialized algorithm to detect common CNVs from extremely high-resolution multi-sample aCGH data. MGVD showed high sensitivity and a low false discovery rate for a simulated data set, and outperformed most current methods when real, high-resolution HapMap datasets were analyzed. MGVD also had the fastest runtime compared to the other algorithms evaluated when actual, high-resolution aCGH data were analyzed. The CNVZs identified by MGVD can be used in association studies for revealing relationships between phenotypes and genomic aberrations. Our algorithm was developed with standard C++ and is available in Linux and MS Windows format in the STL library. It is freely available at: http://embio.yonsei.ac.kr/~Park/mgvd.php.

## Introduction

Copy number variations (CNVs) are a type of the human genomic structural variation. CNVs are now recognized as a major source of human genetic variability, occupying a larger proportion of the genome than single nucleotide polymorphism (SNP) [1]. CNV is causative of various genetic diseases including cancer, and therefore the majority of previous association studies have focused on domains related to cancer [2], [3]. However, CNVs in normal human genomic structure also should be analyzed because CNVs can exhibit different phenotypes in different ethnic groups, sexes, or even family groups. The human germline has been shown to possess copy number variations despite a normal phenotype [4]. Although the mechanisms and medical relevance of CNVs in the human genome are not yet fully understood, a recent study focused on the relationships between CNVs and genes as well as SNPs and genes [5].

Various CNV detection algorithms have been proposed in the past. [6] proposed a circular binary segmentation (CBS) algorithm that is one of the best performing algorithms with high accuracy. However, CBS has high time complexity and was mainly designed for cancer cell analysis. [7] provided a sensitive and robust analytical approach for detecting CNVs but it was also designed for cancer cells analysis with a simple threshold to determine putative CNVs. Few algorithms have been designed for CNV detection in normal human genomes [8], [9] although the importance of CNV in normal human variation has been confirmed in 2004 [10].

The resolution of most cancer and normal data used to be low. The length of CNV is able to be several kilo base pairs or less than it, which can be composed of only one probe in low-resolution data while several probes in high-resolution data. Therefore, algorithms that were designed to find CNVs based on these low-resolution data were only suitable for identifying large-sized CNVs. The currently known common human CNVs are likely smaller than previously thought [11]. Therefore, CNV detection algorithms have to be modified to locate small-sized CNVs. In recent years, several algorithms have been proposed for higher resolution data. An integrated hidden Markov model was designed by [12] for high resolution SNP genotyping data at a kilo-level resolution. However, this algorithm is not suitable for aCGH data which are even higher resolutioned.

One of the best solutions for detecting small-sized CNVs is based on high-throughput, short-read sequence data [13], [14], [15]. However, personalized, high-throughput sequencing is still experimentally costly. Moreover, short read alignment-based approaches to detect CNV require extensive coverage, such as more than 10 coverages, which can increase the cost further. For example, 1000 genome project carried out whole genome sequencing with from 2 to 4 coverages for 179 individuals [16], which is not sufficient to detect CNVs. And most of the approaches to detect CNVs with short-read sequence use an alignment method, which requires long computational time in building index and matching to reference. Alternatively, a few fast and efficient methods for aCGH have been published [17], [18], [19]. A more practical solution to detect small-sized CNVs is to shorten the length of each probe, in other words, to increase the resolution of the aCGH. The Wellcome Trust Sanger Institute recently published high-resolution aCGH data. To generate this data, the institute used 42 million probes spread across 2.1 million probe arrays, with an average probe length of 50 bp. The Wellcome Trust Sanger Institute published normalized intensity data and the additional information for this dataset (http://www.sanger.ac.uk/humgen/cnv/42mio/). All CNVs can theoretically be detected as long as the length of each probe is less than 1 Kb, because CNVs are defined as the gain or loss of size fragments that are greater than 1 Kb in length [20]. Most existing CNV detection algorithms cannot be applied to this high-resolution aCGH dataset without modification, primarily because of their unfeasibly long runtimes.

In aCGH data, the relationship between signal intensity and genomic copy number is not always linear and can vary widely according to the total DNA dosage [21]. To detect true CNV signals, frequent random noise should be excluded and the experimental error of the microarray should also be removed. This is one of the limitations of aCGH data compared to sequencing data. In particular, oligonucleotide-based high-resolution DNA microarray are known to have a low signal to noise ratio (SNR) [22]. Although a few of the existing CNV detection algorithms are robust to error, they are still best suited for analyzing low resolution data.

The aCGH data also has fundamental limitations because of its experimental principles. In aCGH, genomic comparative hybridization is performed using two human genomes: a reference sample and a test sample. The reference sample most commonly used for aCGH up to this point is NA10851, which is assumed to be normal. However, if a reference sample contains genomic variations, then the ratio of the genomic hybridization cannot be measured accurately. For example, if the reference sample has a copy number loss, then the test samples would be reported as having a gain in this area. In general, it has been assumed that the reference sample reflects the absolutely normal and standard status. Recently, copy number information from the reference sample was directly used to consider the abnormality of the reference sample [23]. By aligning short read fragments of the NA10851 reference to a human reference genome (hg18), the reference copy number status was inferred, and this was used to determine the final CNVs. However, to detect real CNVs, the authors used only 1007 candidate CNV areas from 70 individuals composed of 42 M high-resolution aCGH results for 40 individuals [24] and 24 M aCGH results for 30 Asian individuals. An algorithm that takes multiple samples and the effects of a reference sample into account simultaneously has also recently been proposed. This new, multiple sample-based approach, uses a sparse Bayesian prior and expectation maximization algorithm to fit the model [25].

**Figure 1:**
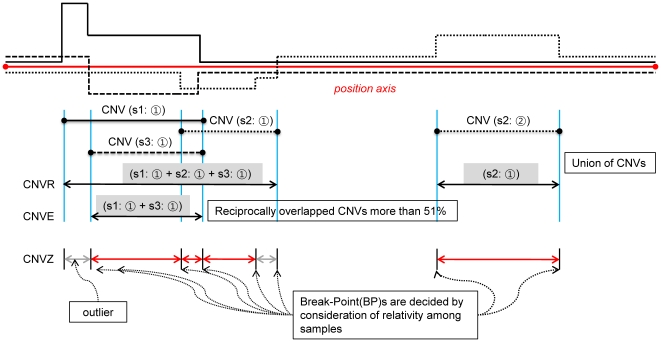
The conceptual difference between CNVR, CNVE, and CNVZ. For three samples, the early methods detected all of the CNVs for three samples individually. The CNVR or CNVE were determined after the CNVs were detected. The CNVR is the union of overlapping CNVs [20] and a CNVE exists if the CNVs reciprocally overlap by more than 51% [23]. In this Figure, “CNV (s2: 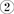)” represents the second CNV of sample 2. The CNVZ is not built from individual CNVs. The start and end positions of the CNVZ are determined by the breakpoint (BP) of the segment, which is identified from the consistency of all the samples. We defined a CNVZ as a locus where the consistency of the log ratio across all of the samples was broken and where samples that were highly positive or negative were present.

It is increasingly important to investigate multiple human genomes for determining genetic variations between some groups, including different ethnic groups. There are algorithms that have been used to detect common CNVs using a statistical frame work [26] [27]. [26] adopts a two-step strategy that calls CNVs for individual samples prior to cross-sample analysis. The recurrent CNV calling approach [28] [29] [30] [31] has been criticized based on the fact that the focus of this approach is the analysis of high-resolution tumor-related data. Recently, whole genome association studies have been carried out. It is essential to identity common CNV which is altered simultaneously across multiple samples for those association studies. We defined this common CNV as CNVZ (CNV zone). CNVZ is slightly different from the CNV region (CNVR) which is an area of CNVs that overlap at least by 1 bp among all the test samples. We considered multiple samples to be a group and analyzed the signals from all of the samples, and then reported the identified CNV sites as CNVZs. In other words, a CNVZ is an area that has a genomic aberration and is determined not just by the simple union of the CNVs detected in each sample. The main conceptual difference between CNVR and CNVZ is the way that the boundary of the copy number loci is defined. Conceptually, a CNVZ is a subset or superset of a CNVR. Figure 1 shows the differences between CNVR, CNVZ, and CNVE, as defined by [24].

In this study, we propose a novel, multi-sample-based, genomic variation detector (MGVD) that successfully handles the huge complexity associated with high-resolutions and the limitations of aCGH. The proposed method identifies CNVZ using multiple samples as a whole, and identifies CNVs from the CNVZs in contrast to existing CNV detection methods. The key features of MGVD are that: 1) it can be applied to both high-resolution and low-resolution data with a reasonable runtime, 2) it directly provides CNVZs throughout the human genome by variation analysis, 3) it has a low false positive and negative rate, as compared to the other algorithms used for analysis of high-resolution aCGH data, and 4) it can be used to determine aberrations of the reference sample by comparative assays of all test samples.

## Results

MGVD targets high resolution aCGH data, including the data published by the Wellcome Trust Sanger Institution, which is composed of 20 European females and 20 African females. This data consists of 42 million probes of whole chromosomes, and is augmented approximately 1,600 times of the first 270 array-based HapMap samples. The machine we used for our experiments comprised an AMD Phenom™ II X2 545 processor, 3.0 GHz, 4 GB RAM and a 32-bit Windows 7 operating system. MGVD was implemented in C++ with STL in Visual Studio 2005. To determine the accuracy and runtimes of the existing algorithm, simulated data created by [32] was used. However, this data was of very low resolution compared to real high-resolution data. Because MGVD is specialized for high-resolution data, the available simulated data is not adequate for testing MGVD. We therefore did not use Willenbrock's simulated data to estimate the optimal parameters or compare our algorithm with other algorithms; however, we did use Willenbrock's simulated data to prove that MGVD works well, regardless of the resolution of the data. Capturing the features of high resolution data and simulating the effects of noise are some of the challenges associated with simulation of high-resolution data. Furthermore, validation techniques for high resolution simulated data have not yet been established. For most of the comparisons we made, we therefore used real data - 40 HapMap samples from the Sanger Institute and the first Korean genome, AK1, published by the Genomic Medicine Institute-Seoul National University. And we also used aCGH data from 30 normal human Asian genomes [23]. The AK1 and 30 Asian aCGH dataset are available at: http://www.gmi.ac.kr/.

### Performance of MGVD with high-resolution aCGH data

MGVD has two main parameters, *θ_dist_* and *θ_cnvz_*. These two parameters were used as the cutoff threshold in each phase noted in the Method section of this paper, and the optimal values for the two parameters were obtained using a repetitive permutation test. However, each chromosome had a different mean and standard deviation of the log ratio profile and different fluctuations, indicating that it is not reasonable to apply one static threshold to all chromosomes. We therefore used optimal parameter values for each chromosome that we determined by comparing the results of our experiment with the validated results of [24].

The biologically validated results presented in [24] were based on 40 high-resolution array CGH samples. In this study, the areas of overlapping CNVs were merged into a CNVE if they had at least a 51% reciprocal overlap. [24] detected 11,700 CNVs that were greater than 443 bp, and 8,599 of these CNVEs have been validated independently. The preliminary false discovery rate was ∼20%, which was regarded as the algorithmic false rate in [24]. We also used these 40 high-resolution datasets for MGVD. We inferred the performance of our algorithm from the validated results based on the assumption that the 8,599 validated CNVEs represent the true answer set.

However [24], used the genomic alteration detection algorithm (GADA), which was proposed by [19] to detect CNVs and biologically validated the found CNVs. Therefore, these validated results are contingent upon the GADA. To overcome this limitation, we compared MGVD with other algorithms using the 40 sample high-resolution dataset and also compared MGVD and GADA with a simulated dataset and AK1 high-resolution aCGH data. Details of these two experiments are provided in the next sub-section.

For performance comparison of the algorithms, we calculated the *false discovery rate* (*FDR*) and *sensitivity* for each chromosome. The answer set was dependent on the dataset used. We defined these measurements, as follows:
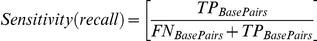


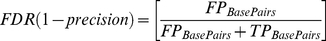
where True Positive (TP) are called when the base pairs of identified CNVZs overlap with the base pairs of the answer set, and False Positive(FP) are called if the base pairs of identified CNVZs do not overlap with the base pairs of the answer set. The true base pairs that are not detected are False Negative (FN). The base pairs for which no CNVZs are detected and there are also no copy number variants present in the answer set are True Negative (TN). The distinguishing feature of our measurements is that they are calculated using base pairs. Most previous methods used the number of CNVs that overlapped by more than 50% with one of the answer CNVs. However, by using a base pair approach, both the *sensitivity* and *FDR* of the algorithm can be calculated precisely, because only the overlapping base pairs between the CNVZ and the answer set are considered. For example, if the CNVZ and the true copy number of the answer set overlap by more than 50%, we do not determine it as 1. We calculate the amount of overlapping base pairs between them. *FDR* is the proportion of falsely detected base pairs of MGVD compared to the entire number of base pairs detected by MGVD. *Sensitivity* is the proportion of base pairs that are truly detected by MGVD as compared with the true answer set, i.e. CNVE. CNVZ and CNVE are conceptually similar. Both indicate relatively common aberrant areas across multiple samples.

**Figure 2:**
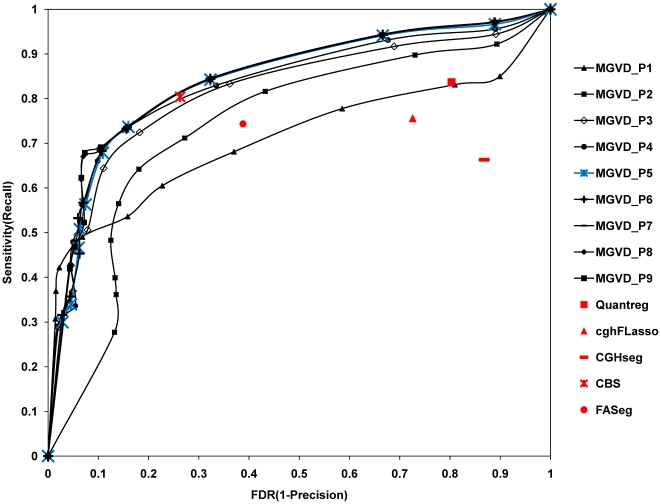
PROC operational curves with 90 parameter sets for real high resolution data for chromosome 22. By varying two parameters of MGVD, 90 different results were generated. Results for the five existing algorithms were obtained using default parameters. MGVD_P5 contains the best parameter set.

The experimental results of repetitive permutation tests to obtain optimal values for the two parameters, *θ_dist_* and *θ_CNVZ_*, for chromosome 22 are shown in Figure 2. In Figure 2, the two parameters that control the trade-off between *sensitivity* and *FDR* are adjusted in MGVD by precision versus recall operation curves (PROC). The experimental results obtained for five existing algorithms using their default parameters are also shown in Figure 2. In Figure 2, the lines from MGVD_P1 to MGVD_P9 indicate the results for each parameter set. Each parameter set, e.g. P1, P2, etc., is composed of a constant *θ_dist_* value and 10 different *θ_CNVZ_* values. For example, P1 has a value of 0.025 for *θ_dist_* and 10 values from 0.05 to 1.4 with intervals of 0.15 for *θ_CNVZ_*. There were nine parameter sets in total. We therefore carried out a total of 90 different experiments by changing each parameter.

**Figure 3:**
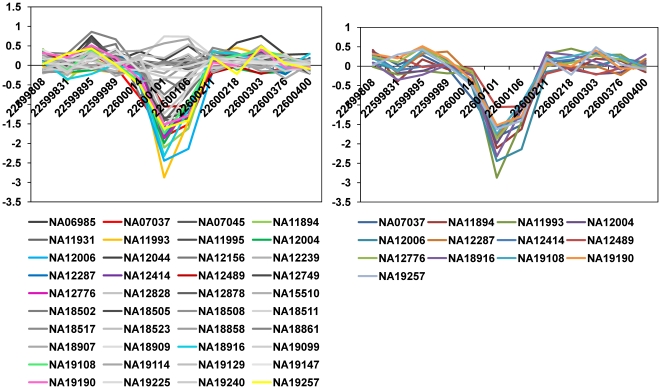
An example of the CNVZ results obtained chromosome 22 using optimal parameters. The graph shown in the left-panel indicates the raw log ratio profile pattern of CNVZ. In the left-panel and right-panel, the colored lines indicate the samples that participate in the CNVZ. The right-panel only shows the samples that participate in the CNVZ. Among 40 samples, 13 samples are turned out to be CNVs in this result.

For chromosome 22, the best parameter set was 0.5 and 0.075 for *θ_cnvz_* and *θ_dist_*, respectively. We found 412 CNVZs and determined that their average length was approximately 8,410 bp using the optimal parameter set. The largest CNVZ was 454,258 bp, and the smallest one was 506 bp. An example of one of the CNVZ results is provided in Figure 3. This CNVZ starts at position 22,599,808 and ends at position 22,600,400, and 13 samples participate strongly in this CNVZ.

### Performance comparison with 7 algorithms

We compared MGVD with the seven algorithms implemented in CGHWeb [33], namely CBS [6], FASeg [34], cghFLasso [35], CGHseg [36], Quantreg [37], GLAD [38], and BioHMM [39]. We ran these algorithms on chromosome 22 only, as this is the smallest chromosome, because all seven algorithms terminated after several days when run on data from all chromosomes. For each algorithm, we built CNVEs from the CNVs identified from the 40 samples. For MGVD, we did not build the CNVE, but, instead, we used the CNVZ. The results are shown in Table 3. The GLAD and BioHMM terminated after several days, even when data from chromosome 22 only was used. Therefore, we were not able to calculate the *FDR* and *sensitivity* for these two methods, which is indicated by non/applicable (N/A) in the figure legends. When we considered *FDR* and *sensitivity* together, then MGVD generally outperformed the other algorithms. MGVD also had an outstanding run time compared to the other algorithms. MGVD processed 502,117 probes approximately 6-times faster than the fastest of the other seven algorithms with a better *FDR* and *sensitivity*. Furthermore, even through MGVD had the lowest *FDR*, it did not have the lowest *sensitivity*. A low *FDR* indicates that MGVD identified most of the CNVEs that were detected by the Sanger Institute.

**Figure 4:**
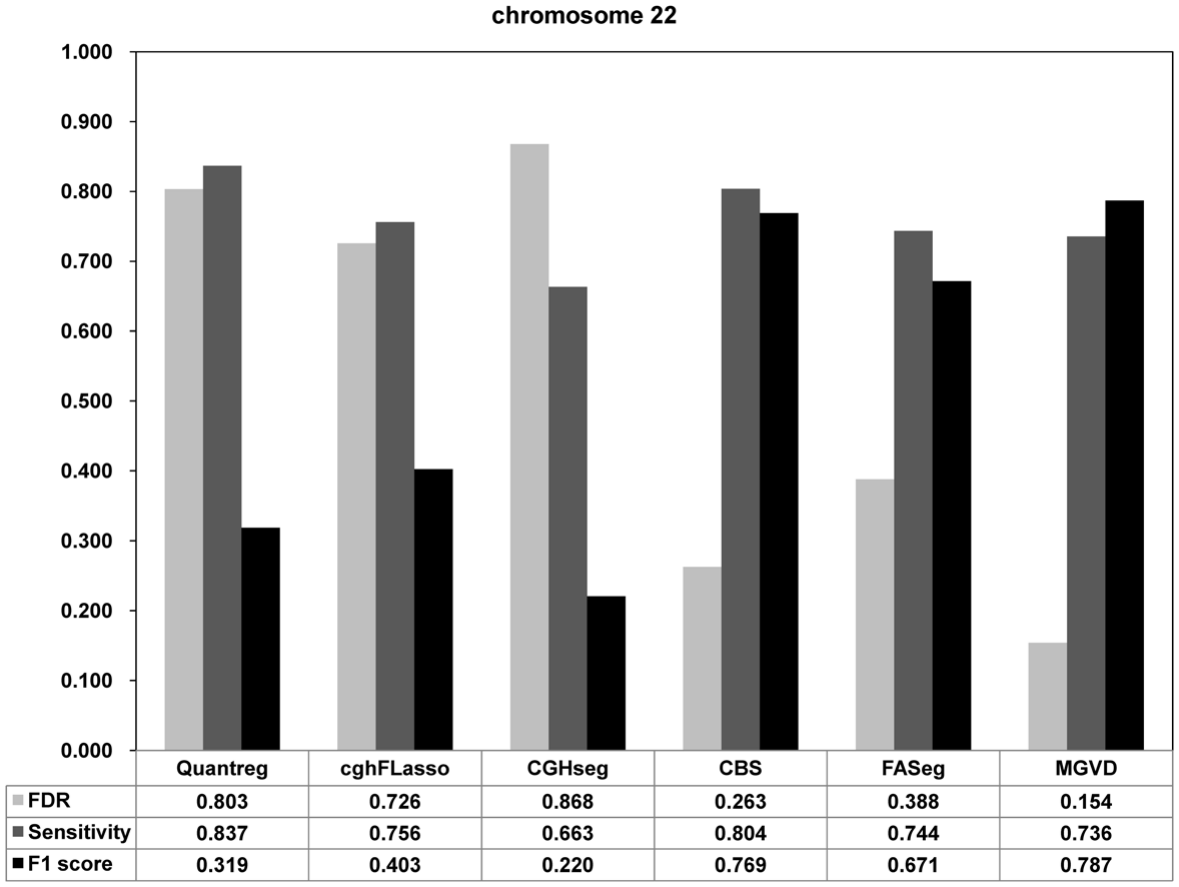
F1 score of five comparing algorithms and MGVD for chromosome 22.

**Figure 5:**
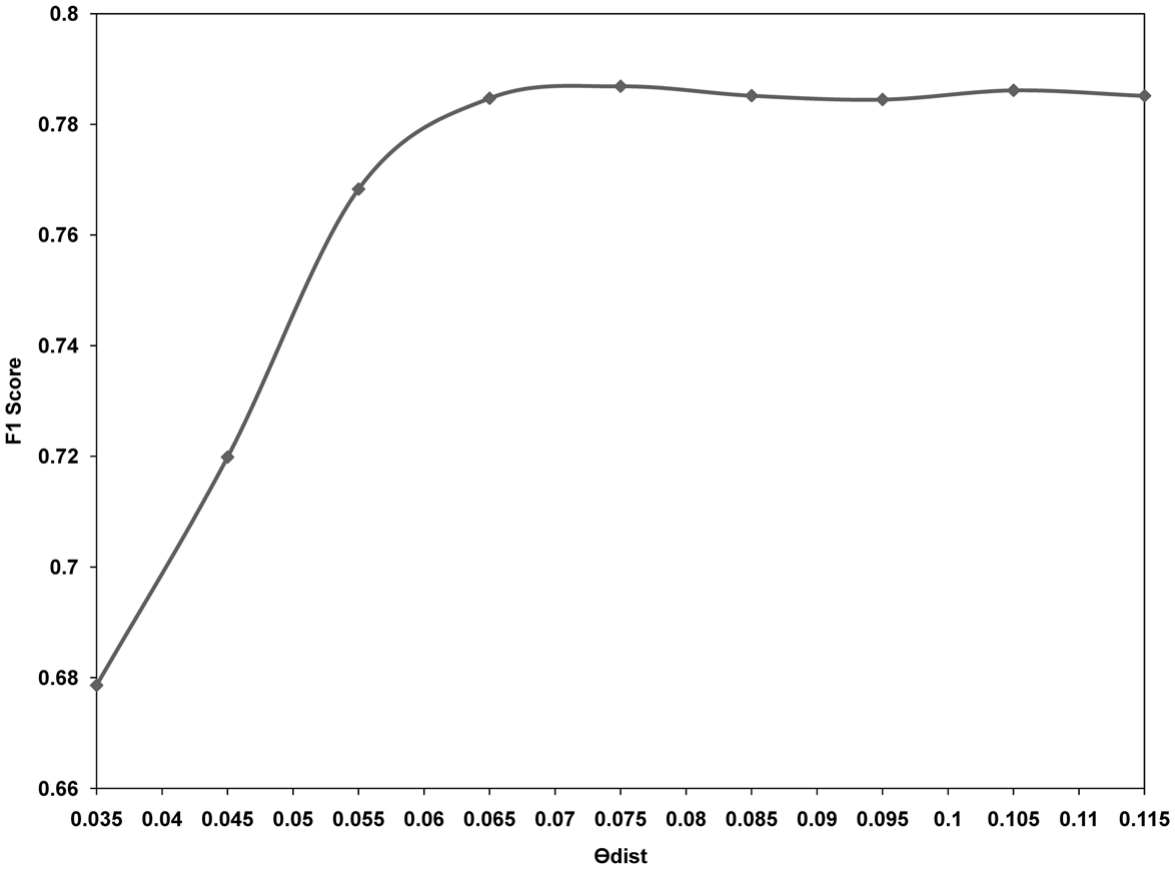
Maximum F1 score of MGVD for chromosome 22 according to various values of the two parameters, *θ_dist_* and *θ_CNVZ_*.

While CNVs are known to cover 12% of the entire genome [20], the CNVEs that the Sanger Institute identified cover much less, approximately 4%. Therefore, the low *sensitivity* and high *FDR* of our algorithm are reasonable according to the Sanger Institute findings. The low *sensitivity* and high *FDR* values that we obtained for our algorithm indicates that it detected aberrant regions that were not identified by [24]. However, these results do not guarantee convergence to the correct answer, because a high *FDR* can indicate an absolutely incorrect result. To compare the performance of our algorithm with the other algorithm, we assumed that the results obtained by [24] were the true results and evaluated the F1 score, which is a measure of accuracy. Figure 4 shows the F1 score, *sensitivity* and *FDR* of MGVD and five other algorithms for chromosome 22. Default parameters were used for five other algorithms. The F1 score of MGVD was the best among the six methods evaluated when the optimal parameters of *θ_dist_* = 0.075 and *θ_CNVZ_* = 0.5 were used. Figure 5 shows the maximum F1 score when we changed the *θ_CNVZ_* and fixed the *θ_dist_* to the value of the x-axis. In File S1, Table 1, we present the optimal parameter set and its F1 score and the quantitative findings for each chromosome. We also present the experimental results of MGVD and the five other methods for chromosome 21 in Figure S4.

**Table 1:** Comparison of the performance of MGVD with those of other algorithms.

Input data	Chromosome 2240 samples	502,117probes covering an average of 50 bp/probe.File size of raw 40 samples is about 205 MB(35,161,372 bp per sample).				
Algorithm	Parameter settings	Average runtime per sample(sec)	*FDR*(%)	*sensitivity* (%)	Identified quantity (bp)	Identification ratio (%)
MGVD	*θ_dist_* = 0.075, *θ_CNVZ_* = 0.5	4.72	15.40	73.55	3,476,988	9.88
Quantreg	λ = 1	25.06	80.32	83.67	24,793,052	80.32
cghFLasso	FDR = 0.05	240.26	72.57	75.61	15,448,348	72.57
CGHseg	Km = 20, S = −0.5	424.72	86.79	66.33	31,115,147	86.79
CBS	α = 0.05	506.67	26.27	80.39	4,733,899	13.46
FASeg	σ = 0.025, δ = 0.1, SR = 50	1074.84	38.79	74.35	7,015,852	19.95
GLAD	Qlambda = 0.999	N/A	N/A	N/A	N/A	N/A
BioHMM	Use clone distances	N/A	N/A	N/A	N/A	N/A

### Performance comparison with GADA

We compared our results with those obtained using GADA, which is one of the fastest and recent CNV detection algorithms with a high accuracy. We used a console version of GADA implemented in standard C that was updated on February 11, 2008. We compared the *FDR*, *sensitivity*, and computational runtime of the different CNV detection algorithms. Because [24] used GADA for CNV detection before validation, the *FDR* and *sensitivity* comparisons with our algorithm are highly dependent on GADA. Therefore, we were not able to use the 40 high-resolution samples for the *FDR* and *sensitivity* comparisons, but were able to make runtime comparisons. To compare the *FDR* and *sensitivity* of our algorithm and GADA, we ran both algorithms on the simulated dataset used by [32] and a Korean individual genome published by [40].

The simulated genome was constructed using statistical information, such as the mean and standard deviation of the log ratio profiles, and had several breakpoints with various lengths. However, this simulated genomic data is shorter than real data and does not include various complex CNV patterns that appear in the real high-resolution aCGH data.

A Korean individual genome, known as AK1, was published recently, representing the first whole genome analysis in Korea and the fifth whole genome analysis in the world. To analyze the AK1 genome, SNPs and CNVs were detected using BAC sequencing and Agilent 24 M aCGH with Illumina BeadStudio 3.1 software.

We used the parameters reported in [24] when we applied GADA to the simulated and AK1 datasets. We used parameter values of *θ_dist_* = 0.045 and *θ_CNVZ_* = 0.57 when running MGVD on the simulated data, and *θ_dist_* = 0.08 and *θ_CNVZ_* = 1.35 when running MGVD on the AK1 data. Because MGVD was more sensitive than GADA to the input data, we determined the best parameter sets for these two data type. For each sample, we built CNVRs from the CNVs GADA detected. The results are presented in Table 4. MGVD and GADA had similar *FDR* values and detected similar numbers of CNVs for the simulated dataset. The *sensitivity* of GADA was higher than that of MGVD, and overall, GADA showed better performance than MGVD for the simulate dataset. For the AK1 datasets, GADA showed high *sensitivity* and *FDR*. However, it detected 98% of the whole genome as CNVs while MGVD detected only 3.4% of the whole genome as CNVs. When we adjusted the parameter set of MGVD, the aberrant genomic quantity became similar to that in the answer set. However, it was not possible to find an optimal parameter set for GADA that provided a similar genomic quantity to the answer set. We carried out several experiments changing the parameters in GADA. However, the identified genomic quantity was similar throughout these experiments. GADA was not sensitive to changes in the values of various parameters. As a result, the F1 score of GADA was about 10 times worse than that of MGVD for the AK1 data.

**Figure 6:**
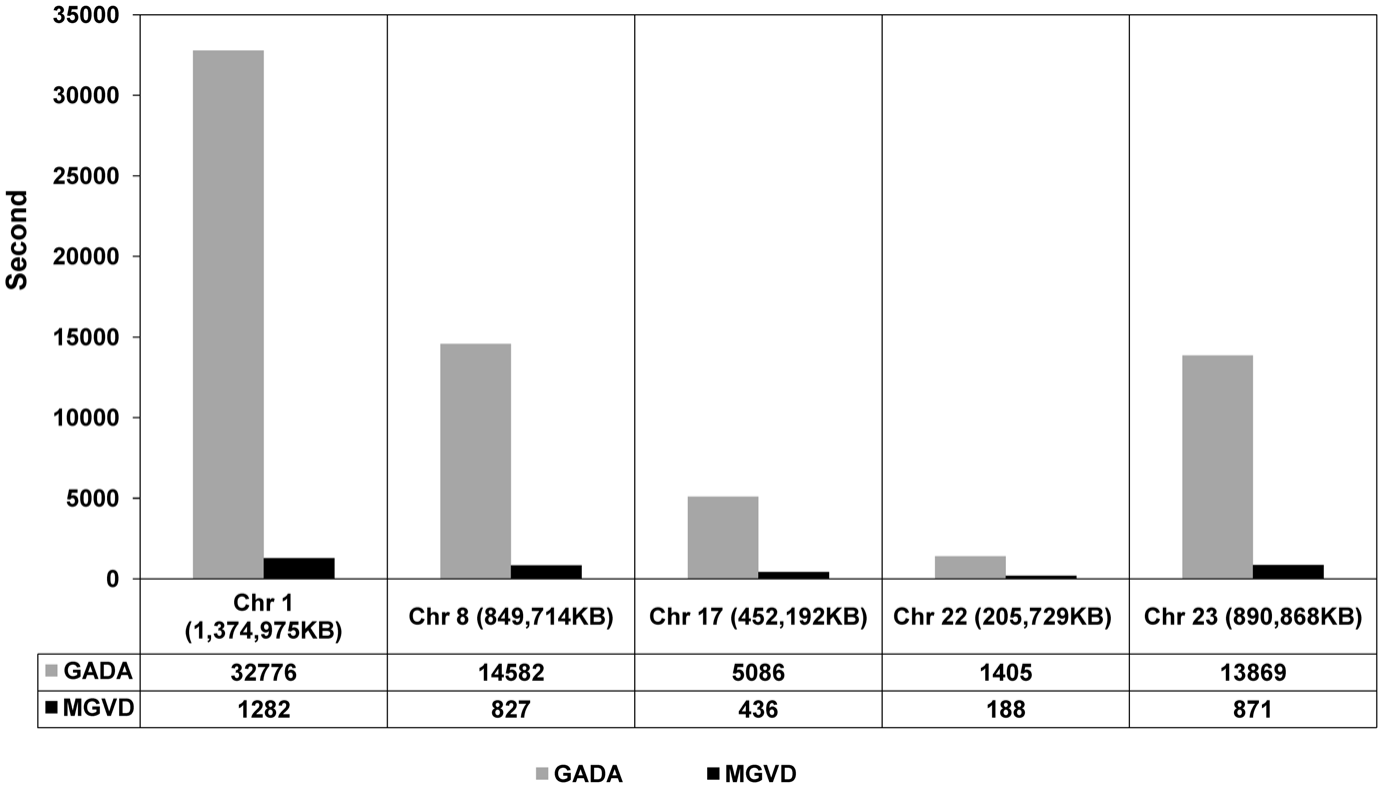
Comparison of the computational runtime of MGVD and GADA for five chromosomes. For each chromosome, data from 40 samples was available. We measured the total runtime with 40 samples for each chromosome. The runtimes of both MGVD and GADA increased linearly as the data size increased. For all five various sized chromosomes of various sizes, the MGVD runtimes were shorter than the GADA runtimes.

The validated CNVs for the AK1 dataset cover approximately 2.75% of the entire genomic quantity of chromosome 22. This is a relatively small portion of known CNVs and CNVE. The reason for this is that the number of validated CNVs depends on the algorithm or method that was used. The AK1 dataset was also tested using 42 M aCGH data, but these results have not yet been fully validated. There could be true CNVs that were not detected and validated by previous studies. Furthermore, MGVD was practically more feasible than that of GADA despite the low number of validated CNVs that this algorithm yielded. Figure 6 shows a comparison of the computational runtimes of MGVD and GADA for real chromosomes of various sizes.

### Performance comparison with CMDS

We also compared the MGVD results with those obtained using CMDS [28], which was recent recurrent CNV detection algorithm. Recurrent CNVs mean the common CNVs across multiple samples. Both MGVD and CMDS were designed to identify common CNVs; however, CMDS did not work on 42 M high resolution aCGH data. Therefore we used the 24 M resolution aCGH data for 30 Asian published by [23]. We compared the performance of MGVD and CMDS in terms of *FDR*, *sensitivity* and F1 score. The CNVE results for the 30 Asian aCGH dataset were reported by [23] and used these results in our performance tests. CMDS worked with default parameter set: block size of 50, cutoff of 10, step size of 1. We also determined the optimal parameters of MGVD. When we used *θ_dist_* = 0.07 and *θ_CNVZ_* = 1.0, the identified genomic quantity was similar to that of the CNVE results for the 30 Asian samples. The F1 score of MGVD was relatively better than that of CMDS. The results are presented in Table 5. However, neither of the F1 scores obtained was very high. This is because only 116 preselected CNV regions of [23] were biologically validated with qPCR. The compassion results between MGVD and CMDS are presented in File S2.

### Average runtime of MGVD

**Figure 7:**
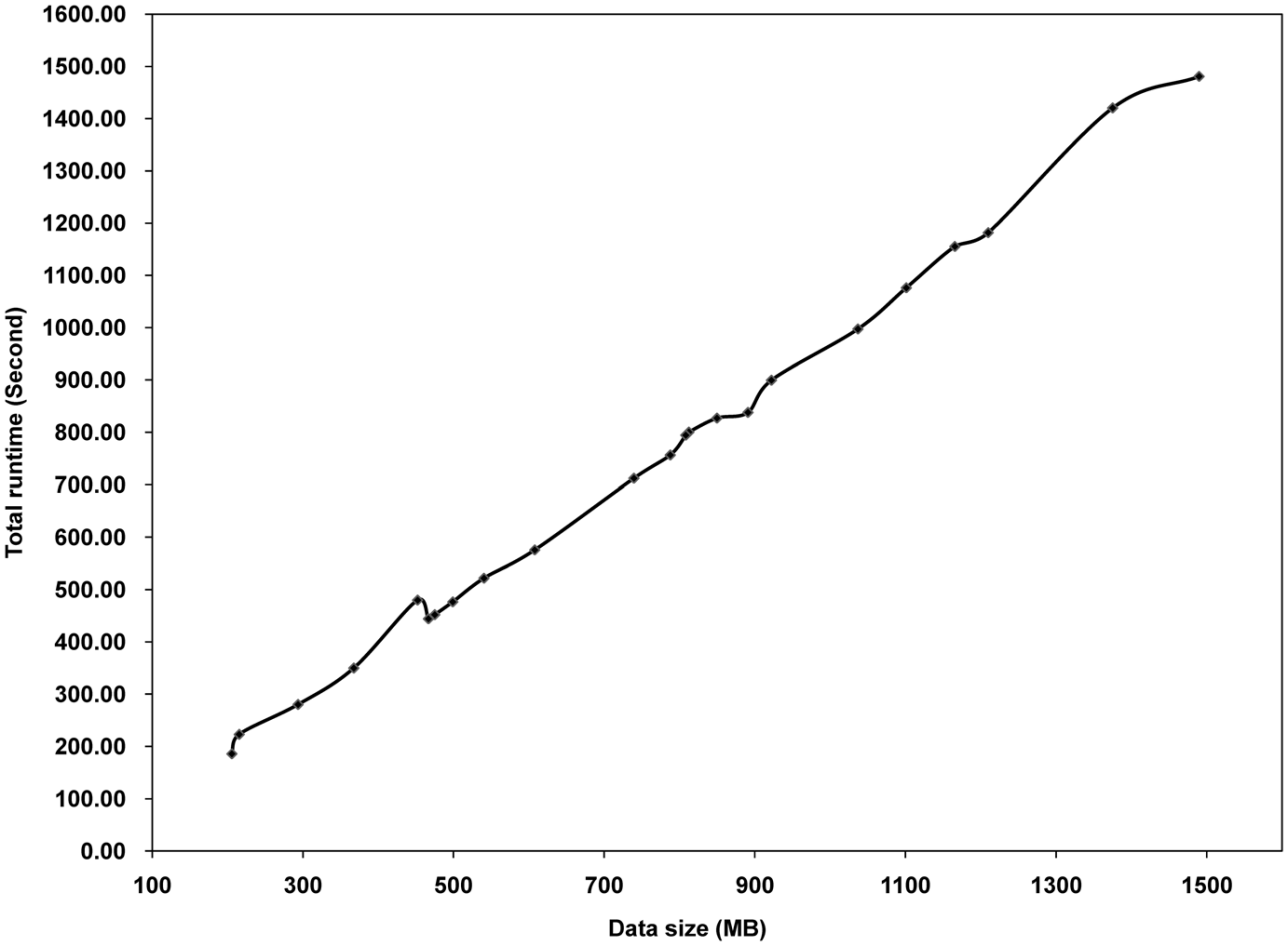
Runtime of MGVD for 40 high resolution aCGH samples. The entire runtime increased linearly with increasing data size. During the runtime, MGVD generated CNVZs and CNVs for each sample.

We ran MGVD using whole chromosomes and obtained a practical runtime for analysis of 42 M high-resolution aCGH data. The runtime for each chromosome was derived from the optimal threshold test, which involved 100 iterative permutation tests for two parameters. We obtained 100 different runtimes for four phases and then took the average; the results are shown in Figure 7. The average runtime for each chromosome was measured using 40 high-resolution aCGH datasets. The runtime was linearly increased according to data size. All CNVZs and CNVs result for whole chromosomes are presented in File S3.

## Discussion

In this study, we developed to devise a new method to identify CNVZs and CNVs across multiple high-resolution aCGH data sets. We proposed a new concept, namely a common CNV occurring area, which we called a CNVZ. Current methods detect CNVs in various samples and then build common copy number regions from all of the identified CNVs. However, biological studies often require common CNV patterns detected in multiple samples, especially when analyzing the normal human genome. No suitable methods have been developed for this purpose, at least not of which we are aware. By comparing all samples at a certain locus, MGVD determines whether that locus is a genomic aberration or not. Our experimental results demonstrated that MGVD had an attractive computational complexity, compared with the GADA, which is one of the fastest algorithms currently available. The time complexity of the current methods is proportional to the number of samples, while this is not the case for MGVD. The *sensitivity* and *FDR* of MGVD were also comparable with those of several other well-known algorithms. We conducted experiments using three real datasets (the 40 high-resolution aCGH samples, AK1 data, and Asian 30 aCGH datasets) and one simulated dataset. MGVD is faster than the current methods when a large number of samples are analyzed because it uses a clustering approach, which is particularly advantageous when detecting CNVZs using ethnic group data. MGVD also accepts data from various platforms. For example, MGVD can be applied to high-throughput sequencing data. To detect CNVs using sequencing data, short reads have to be aligned to the reference genome sequence, and then a series of alignment scores can be obtained, similar to high-resolution aCGH data.

## Materials and Methods

**Figure 8:**
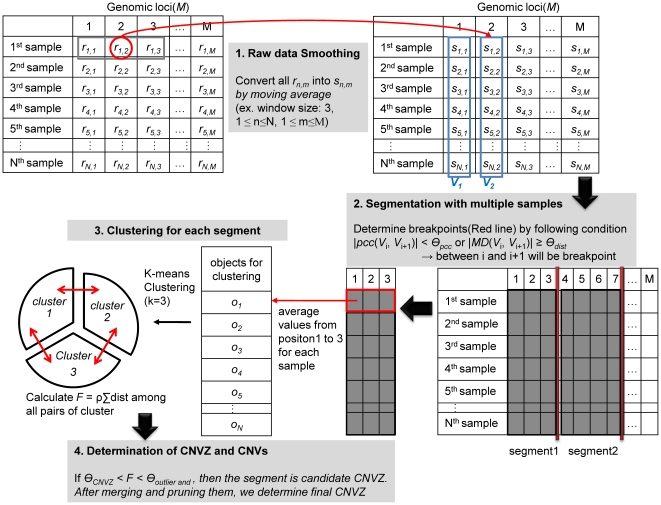
Schematic Process Flow Chart of MGVD for high resolution aCGH data. MGVD consists of four phases which are sequentially executed. We find candidates in advance through phases 1 and 2. Then we get the final results through phases 3 and 4.

MGVD was designed to achieve high performance, i.e. high accuracy and low runtime complexity, when analyzing multiple, large, high-resolution aCGH datasets which has a low SNR, in order to identify CNVZs across all samples at once and to identify CNVs for each sample. MGVD consists of four major phases: (1) smoothing the raw high-resolution aCGH data by using a moving average during file loading, (2) making segments, (3) clustering the samples for all of the segments, and (4) determining the CNVZs and CNVs from the clustering results. The schematic process flow chart of MGVD is described in Figure 8.

### Notations

**Figure 9:**
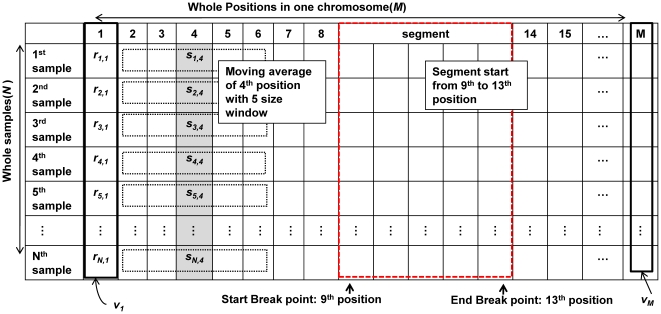
Multi-sample aCGH data and notations used in this paper. Descriptions of the notations used in the paper are provided in Table 1. The left, black-dotted rectangle containing *s_i,j_* indicates the window used for the moving average. The right, dotted, red rectangle indicates a segment.

Table 1 summarizes some of the notations used in this paper and provides brief descriptions of these notations. Detailed definitions of these notations are provided in appropriate locations in the text. Figure 9 shows several basic notations and the format of the real data.

### Raw data smoothing

High-resolution aCGH data consisting of a large number of probes is known to have a low SNR. This results in log ratio signal patterns that are highly fluctuating; detecting genomic variation is therefore difficult. To eliminate these fluctuations, earlier algorithms generally used smoothing methods, such as the wavelet technique. We used a moving average to smooth the log ratio pattern with respect to position for all of the samples simultaneously, because of its simplicity. We identified the optimal window size by evaluating a variety of window sizes. The experimental results for optimal window size are provided in Figure S1. The optimal window size was identified as 11, which is almost equal to the expected minimum size of a CNV, 550 bp. In high- resolution 42 M aCGH data, one probe covers approximately 50 bp. We implemented the moving average during the file loading phase to reduce the overall runtime. This procedure is detailed in File S1, Table 2.

**Table 2:** The *sensitivity* and *FDR* dependence of the algorithms on the data type.

Data	Simulated dataset [32](500 probes×20 samples)		Chromosome 22 of AK1 [40](Nimblegen 42 M high-resolution)	
Answer set	Five CNVs(composed of 92 probes)		28 CNVs (Quantity is 967,462 bp)	
Method	MGVD	GADA	MGVD	GADA
*sensitivity* (%)	89.13	100.00	70.92	98.97
*FDR* (%)	8.88	8.91	41.50	97.17
F1 score	0.90	0.95	0.64	0.06
Identified quantity (bp)	90 probes	101 probes	1,197,547	34,606,907
Identification ratio (%)	18.00	20.20	3.40	98.42
Average length of detected CNVs	8.1 probes	20.2 probes	16,183 bp	96,130 bp

### Segmentation with multiple samples

The purpose of segmentation in our approach differs from that of existing CNV detection algorithms. In most existing algorithms, segmentation is used to find the breakpoints to identify the CNVs for one sample. We used segmentation to identify the points that break the continuous consistency of the log ratio pattern across all samples by comparing *V_i_* and *V_i_*_+1_. If *V_i_* and *V_i_*_+1_ have significantly different log ratio patterns the (i+1) position becomes a breakpoint. Using these breakpoints, *N* X *M* is divided into numerous segments, *SEG_i,j_*, starting from the i_th_ position to the j_th_ position. To achieve this, we computed the absolute value of *Pearson*'s correlation coefficient (PCC) of all adjacent positions. We also simultaneously calculated the *Manhattan* Distance (MD) between *V_i_* and *V_i_*_+1_. These two measurements are defined as follows:
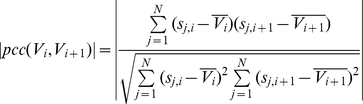


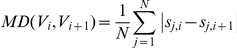
where *s_i,j_* is the smoothed log ratio value, which is defined in Table 1. The reason why we used PCC is to identify the positions that break the consistency with respect to position. If some samples do not follow the log ratio flow of other samples between *V_i_* and *V_i_*_+1_, then the PCC value is close to 0. If the value of PCC is close to +1, then the log ratio pattern between *V_i_* and *V_i_*_+1_ is synchronously up or down. However the sum of the ratio difference can be large, even if PCC is close to +1. For example, if the PCC value of *V_i_* = [0,1,0,1,0,1] and *V_i_*_+1_ = [0,10,0,10,0,10] is 1, the sum of the ratio difference is 27. The position (i+1) of this example has to be the breakpoint because the continuous consistency is broken by the 2^th^, 4^th^, and 6^th^ samples. As a result, the PCC is a necessary condition but not a sufficient condition. Therefore, we also applied the MD to identify segments. Similarly, if the value of PCC is close to −1, some of the cases cannot be broken. For example, when *V_i_* = [4,5,4,5,4,5] and *V_i_*_+1_ = [5,4,5,4,5,4], the PCC value will be −1. However, the actual sum of the ratio difference will not be higher than expected. In this example, the position (i+1) does not have to be the breakpoint. A highly negative PCC also can be used to identify segments with MD. Therefore, we used the absolute value of the PCC and MD to calculate the actual distance sum of *V_i_* and *V_i_*_+1_. To determine whether this point could be broken or not, we used the following determination rule:
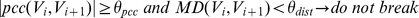


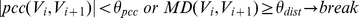
where *θ_PCC_* and *θ_dist_* are the criteria for deciding break-points. While changing the *θ_PCC_*, we carried out experiments to evaluate performance for deciding the optimal *θ_PCC_*. We found that, changing the value of *θ_PCC_* had a little influence on *sensitivity* and *FDR* compared to other parameters. *Sensitivity* is discussed in the Results section. Therefore, we set *θ_PCC_* to 0.8 which indicates present a highly correlated state between *V_i_* and *V_i_*_+1_. The experimental results for *θ_PCC_* are provided in Figure S2. In conclusion, the *N* X *M* was separated into a few sub-matrices called segments, i.e. *SEG_i,j_*, by breakpoints. This procedure is detailed in File S1, Table 3.

**Table 3:** Performance comparison of MGVD and CMDS.

Data	Chromosome 22 of Asian 30 [23](Agilent 24 M high-resolution)File size of the 30 raw samples is about 76 MB(35,136,378 bp per sample)	
Answer set	104 CNVE (6,846,363 bp)	
Method	MGVD	CMDS
*sensitivity* (%)	48.41	14.38
*FDR* (%)	45.50	40.93
F1 score	0.51	0.23
Identified quantity (bp)	6,081,610	1,667,547
Identification ratio (%)	17.30	4.74
Average length of recurrent CNV (bp)	17,376	13,781
Max length of recurrent CNV (bp)	371,559	515,022
Min length of recurrent CNV (bp)	503	928
Runtime (min′ sec″)	1′12″	1′33″

### Clustering for each segment

In this phase, we describe how to decide whether each segment is candidate CNVZ or not. Samples of a segment have a continuous and consistent log ratio. Candidate CNVZs are determined by clustering these samples. Each *SEG_i,j_* is transformed into an object set for clustering. The transformation is as follows:
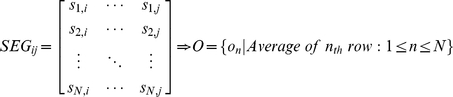
where *O* is the set of *o_n_* which is the average value of the n_th_ row in *SEG*. First, we clustered the average values of each row of the *N* X *j* matrix, each of which represents a sample. If all the elements in the *N* X *j* matrix are used for the clustering, then some of the samples can be included in a cluster of two or more samples, because their elements can be partitioned into different clusters. We decided whether the *SEG_i,j_* was a candidate for the CNVZ or not without taking into consideration multiple clusters for one sample. Then, we carried out *k-means* clustering, which is a fast and typical partitioning-based method.

We assumed that one segment could probabilistically have three states on their samples: gain, neutral, and loss. It might be argued that there are more states than the three that we assumed, such as outlier and high gain states. However, outliers without their own state are removed by *k-means* clustering. Furthermore, high gain or extreme loss states can be included as gain and loss states, respectively. Thus there are only three states in total. Based on this reasoning, we do not need to find the optimal *k* when evaluating the clustering model with AIC or BIC. We therefore fixed *k* to 3. Furthermore, we do not have to allow for non-convex shapes because our objects are in 1-dimensional space. With these constraints, we applied *k-means* clustering to partitioning the segments.

The time complexity of *k-means* clustering is *O(mkt)*, where *m* is the number of objects, *k* is the number of clusters, and *t* is the number of iterations. In our method, the values of *m* and *k* are sufficiently small, and the value of *t* is generally less than that of *m*. We performed clustering for all of the segments. However, because the number of segments, *m*, was much less than the number of all of the positions, *M*, the entire algorithm was executed in a reasonable amount of time. Our *k-means* clustering procedure is detailed in File S1, Table 4.

**Table 4:** Notations used throughout the manuscript.

Symbol	Definition
***N X M***	Raw input data matrix composed of ***N*** samples and ***M*** positions
***r_i,j_***	A raw log ratio of the ***i***_th_ sample in the ***j***_th_ position
***s_i,j_***	A smoothed log ratio of the ***i***_th_ sample in the ***j***_th_ position
***V_i_***	A ***N X 1*** sub-matrix at the ***i***_th_ position in the ***N X M*** matrix
***SEG_i,j_***	A ***N X j*** sub-matrix from the **i**_th_ to the ***j***_th_ position
***CLU_i_***	A set of clusters in the ***i***_th_ segment
***CZ_i,j_***	A CNVZ from the ***i***_th_ to the ***j***_th_ position

### Determination of CNVZs and CNVs

**Figure 10:**
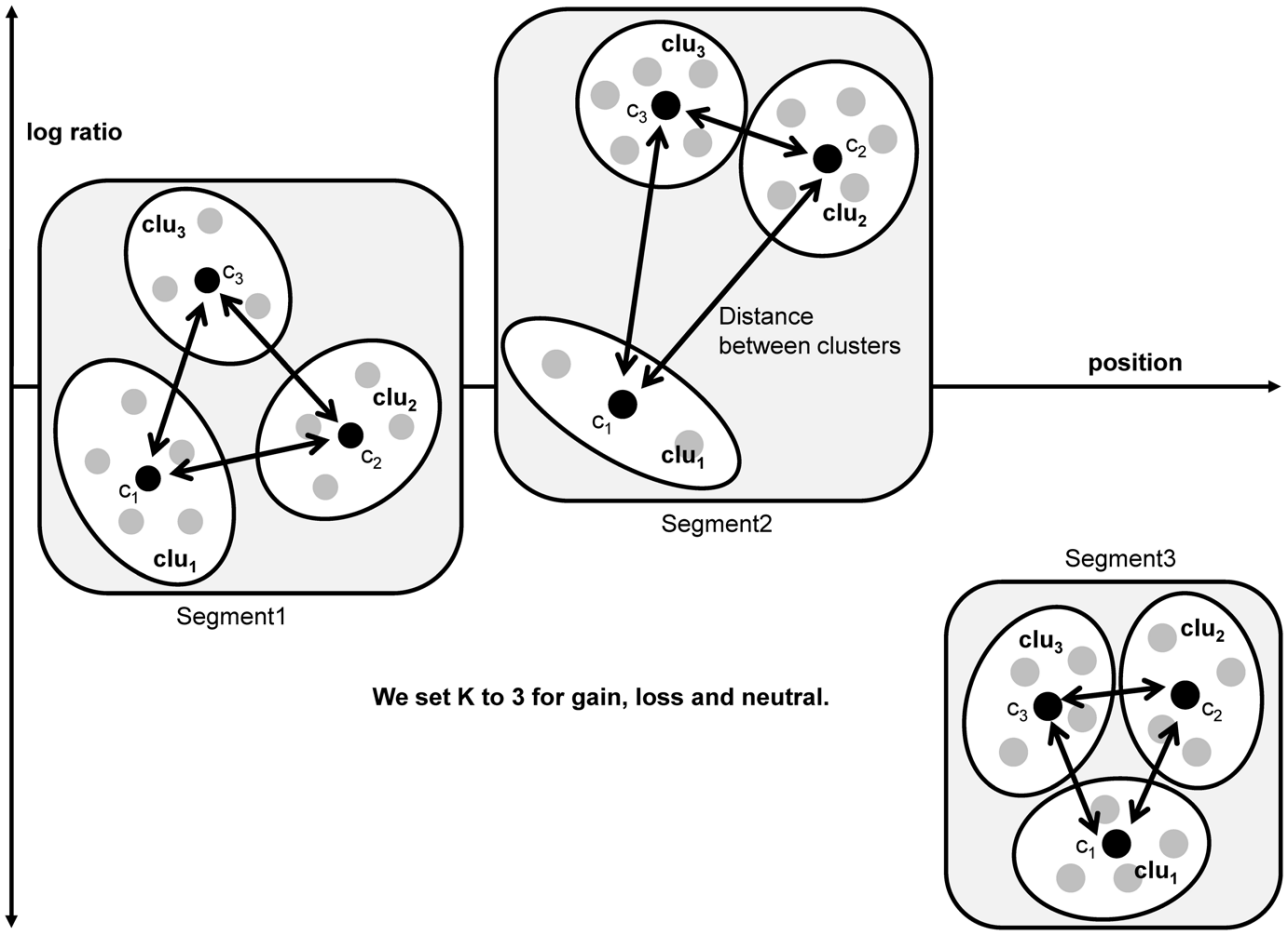
Three possible distributions of clusters. It would be problematic to just consider the distances between clusters. The gray circle indicates the objects in a cluster, and the black circle indicates the centroid of a cluster. This is a conceptual figure to explain the various relationships among clusters.

We identified CNVZs and CNVs by analyzing the distributions of three clusters for each segment. To determine CNVZs, we defined a scoring function that is based on the distance among centroids and can be used to analyze various cluster distributions. Basically, each sample can have a variation or neutral state in a segment. Sometimes, numerous CNVs occur simultaneously across all samples. The scoring function was designed to consider all these possible distributions of clusters in a segment. Figure 10 shows three possible cluster distributions.

We identified whether the region of a segment was an aberration or not by evaluating the distances between all possible pairs of clusters in the segment. For example, if one segment has a large sum of distances between possible pairs of clusters, then these clusters are located far away from each other. In this case, the segment is likely to be judged to be a CNVZ. By using only the distance sum of possible cluster pairs, however, aberrations cannot be detected in some cases, such as in segment 3 in Figure 10. Segment 3 has a small distance among centroids in contrast to the other cases, but its objects have a highly negative log ratio with little variation among the objects; therefore, Segment 3 can be considered a CNVZ. To solve the problem noted above, we used the following the scoring function:
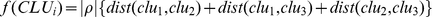
where *dist*() is the distance between two centroids of *CLU*_i_. If these three clusters are close to each other, the sum of distances is small. If the value of *f*(*CLU_i_*) is high, then *CLU*_i_ is determined to be a CNVZ. However, the sum of all of the distances alone cannot cover all of the possible clustering cases. For example, all the clusters of segment 3 in Figure 10 are close to each other, and all the centroids are highly negative. However, according to *f*(*CLU_i_*), segment 3 could be a neutral area, because the sum of all the distances is low. Segment 3 cannot be distinguished from real a neutral area where all the centroids of clusters are located around the zero log ratio profile. Therefore, in this case, the score is weighted to reduce the false rate.

The weight, *ρ*, is the sum of flags, where flag ∈ {1, 0, −1}. Let *μ* and *σ* be the mean and standard deviation of all the centroids for all the segments, respectively. We assume that a cluster whose absolute centroid value is greater than *μ*+*σ* can be considered to be out of a neutral position. According to our assumption, the flag is calculated as follows:
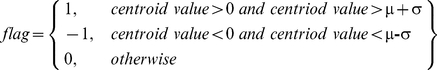
For example, if we assume that a set of centroids of *CLU*_n_ is {−3, −4, −2} and that *μ*+*σ* is 1, then *ρ* will be −3. As another example, if there is a set of centroids that is {−3, −4, 2}, then *ρ* will be −1, because *ρ* is the sum of −2 and 1.

The segment with *θ_cnvz_*<*f*(*CLU_i_*)<*θ_outlier_* is selected as a candidate CNVZ. Here, *θ_cnvz_* is the threshold to determine a candidate CNVZ and *θ_outlier_* is the threshold used to trim outlier segments with a very high value of *f*(*CLU_i_*). One of these two parameters, *θ_outlier_*, is not sensitive to performance if it is larger than a certain value. Because most samples have similar log ratio distributions and outliers have extremely high or low log ratios, we can determine the optimal criterion for pruning outliers. We set *θ_outlier_* to 3.5. The experimental results for *θ_outlier_* are provided in Figure S3.

To estimate the CNVs for each sample, we identified samples that participate in CNVZ. After identifying all the CNVs for each sample, we merged and pruned the CNVs to build the final set of CNVs for each sample. Finally, the candidate CNVZs were also merged and pruned. The adjacent candidate CNVZs were merged into a large CNVZ if their interval was less than 1 Kbp. The segmentation phase of our algorithm identifies short segments, <0.5 Kbp. However, these segments can be disregarded, because the minimum CNV size is approximately 0.5 Kbp in the NimbleGen aCGH dataset. Through these two post processes, the final CNVZs were identified and the segmentation process, which is closely related to the breakpoint decisions, was supplemented. As a result, those processes play a role in maximizing the length and accuracy of CNVZs. The algorithm for determining CNVZs and CNVs is shown in File S1, Table 5.

**Table 5:** Three categories of CNV calls proposed by [23].

Call categories	Case ID	Status of the reference sample	Status of the test sample	Ostensible result/True result	Can be solved by MGVD?
Obscure	case1	Complete Loss	Very high and unstable	CNV/Neutral	*O*
	case2	Heterozygous Copy Number: loss(1 copy), gain	Heterozygous Copy Number: gain, loss	CNV/CNV	*O*
Covert	case3	Copy Number: loss, gain	Copy Number: loss, gain	Neutral/CNV	*X*
Overt	case4	Normal	Copy Number	CNV/CNV	*Not required*

### Consideration of reference copy number

Our approach can identify CNVZs taking into consideration aberrations of the reference sample by using comparative assays for all test samples. A recent study categorized CNV calls into three possible cases: obscure, covert and overt [23]. An obscure call means that the test sample will come out having a copy number but this is caused by an aberration in the reference sample. If the reference sample has a complete loss, the test sample is reported to have a high gain. In this case, however, the test sample should be neutral. A covert call means that the status of the reference and test sample have the same copy number variation, i.e. either both are gains or both are losses, therefore the test sample will end up as neutral. An overt call indicates that the reference sample does not have copy number variation. This is the basic assumption in most studies. Details of these three categories are provided in Table 2.

Of the three categories represented in Table 2, obscure calls can be detected by our approach. Here, overt calls are exceptional because they are a basic assumption of the aCGH platform. It is impossible to call covert CNVs using our algorithm because it does not consider neutral segments, or using the sequencing information from the reference sample. Our algorithm uses only the distribution of clusters to estimate the reference copy number.

Obscure calls are false positive results due to a discrepancy related to the reference copy number. There are two sub-categories of obscure call: one due to complete loss and the other to a heterozygous copy number. The two sub-categories, case 1 and case 2, are shown in Table 2. To detect these cases without reference sample sequencing, we inferred the reference copy number by analyzing CNVZs as follows.

**Figure 11:**
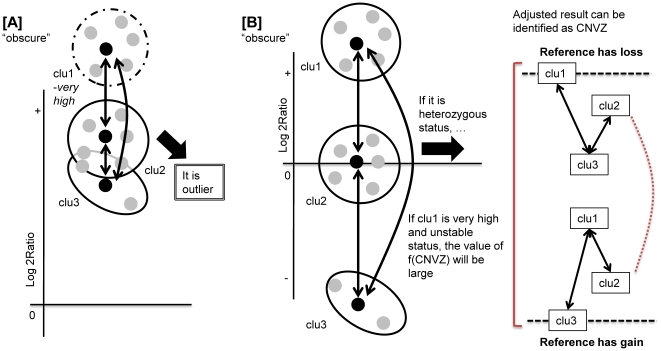
Schemes to consider the reference copy number in MGVD. The left Figure [A] indicates case 1, which has a complete loss in the reference. MGVD detects these cases as neutral. The right Figure [B] indicates case 2, which has a heterozygous copy number compared with the test sample. MGVD detects these cases as CNVZs.

First, if the centroid of one cluster of a CNVZ has a high ratio profile and those of the other two clusters are also somewhat high, the value of *f*(*CLU_i_*) will be large. However, if the value of *f*(*CLU_i_*) is higher than *θ_outlier_*, this CNVZ will be identified as a neutral outlier. We assumed that the appearance of the cluster with a high centroid was caused by a complete loss in the reference. In Figure 11, the left panel notated [A] shows the simple principle underlying our proposed method.

Second, if the three clusters of a segment are widely spread without satisfying the outlier condition, this segment is identified as a CNVZ. In this case, however, the reference sample might be said to have a copy number variation. In our algorithm, if the reference has a heterozygous copy number compared to the test sample, the true result is adjusted. In the example shown in the right panel of Figure 11, the reference sample has a loss, therefore the centroids of the three clusters must be lower and vice versa. Nevertheless, both of these two cases will be identified as CNVZ by our algorithm. When all the clusters of a CNVZ are far away from the medium cluster, whose centroid is nearby zero, that CNVZ will be detected as a CNVZ despite the heterozygous copy number of the reference sample.

## Supporting Information

Figure S1 **F1 score according to window size.** While changing the *window size* from 7 to 16, we also changed *θ_CNVZ_* from 0.4 to 0.85. The graph shows the maximum, minimum and average f1 scores when we change the parameters. The horizontal bar for each *window size* indicates the average f1 score and it was maximal at *window size* 11. Therefore, we decided the optimal *window size* to be 11.
(TIF)

Figure S2 **F1 score according to** ***θ_PCC_*** **.** While changing the *θ_PCC_* from 0.4 to 0.85, we also changed *θ_CNVZ_* from 0.4 to 0.85. The graph shows the maximum, minimum and average f1 scores when we change the parameters. The horizontal bar for each *θ_PCC_* indicates the average f1 score. If we use the large value for *θ_PCC_*, which is close to 1, the size of the segment will increase, while the number of segments will decrease. It may slow down the performance because we have to find small sized CNVZ. Therefore, we decided the optimal *θ_PCC_* to be 0.8 which is clearly distinguished from the previous ones and which is not too close to the larger value, 1.
(TIF)

Figure S3 **F1 Score according to** ***θ_outlier_*** **.** While changing the *θ_outlier_* from 1.5 to 6, we also changed *θ_CNVZ_* from 0.05 to 1.4. The graph shows the maximum, minimum and average f1 scores when we change the parameters. The horizontal bar of each *θ_outlier_* means the average f1 score. If we use a value which is larger than 3.5 as *θ_outlier_*, the f1 score is in equilibrium condition. Therefore, we decide the optimal *θ_outlier_* to be 3.5.
(TIF)

Figure S4 **F1 score of 5 comparing algorithms and MGVD on chromosome 21.**
(TIF)

File S1 **Five supplemental tables.**
(DOC)

File S2 **Comparison results of our method and CMDS using the 30 Asian aCGH datasets.**
(XLS)

File S3 **CNVZ results identified by our method for whole chromosome.**
(XLS)

## References

[1] Levy S, Sutton G, Ng PC, Feuk L, Halpern AL (2007). The diploid genome sequence of an individual human.. Plos Biology.

[2] Berger JA, Hautaniemi S, Mitra SK, Astola J (2006). Jointly analyzing gene expression and copy number data in breast cancer using data reduction models.. Ieee-Acm Transactions on Computational Biology and Bioinformatiocs.

[3] Lee H, Kong SW, Park PJ (2008). Integrative analysis reveals the direct and indirect interactions between DNA copy number aberrations and gene expression changes.. Bioinformatics.

[4] Iafrate AJ, Feuk T, Van Puymbroeck L, Rivera MN, Listewnik ML (2004). Detection of large-scale variation in the human genome.. Journal of Molecular Diagnostics.

[5] Stranger BE, Forrest MS, Dunning M, Ingle CE, Beazley C (2007). Relative impact of nucleotide and copy number variation on gene expression phenotypes.. Science.

[6] Olshen AB, Venkatraman ES, Lucito R, Wigler M (2004). Circular binary segmentation for the analysis of array-based DNA copy number data.. Biostatistics.

[7] Price TS, Regan R, Mott R, Hedman A, Honey B (2005). SW-ARRAY: a dynamic programming solution for the identification of copy-number changes in genomic DNA using array comparative genome hybridization data.. Nucleic Acids Research.

[8] Locke DP, Sharp AJ, McCarroll SA, McGrath SD, Newman TL (2006). Linkage disequilibrium and heritability of copy-number polymorphisms within duplicated regions of the human genome.. American Journal of Human Genetics.

[9] Wong KK, deLeeuw RJ, Dosanjh NS, Kimm LR, Cheng Z (2007). A comprehensive analysis of common copy-number variations in the human genome.. American Journal of Human Genetics.

[10] Wigler M, Sebat J, Lakshmi B, Troge J, Alexander J (2004). Large-scale copy number polymorphism in the human genome.. Science.

[11] Perry GH, Ben-Dor A, Tsalenko A, Sampas N, Rodriguez-Revenga L (2008). The fine-scale and complex architecture of human copy-number variation.. American Journal of Human Genetics.

[12] Wang K, Li MY, Hadley D, Liu R, Glessner J (2007). PennCNV: An integrated hidden Markov model designed for high-resolution copy number variation detection in whole-genome SNP genotyping data.. Genome Research.

[13] Alkan C, Kidd JM, Marques-Bonet T, Aksay G, Antonacci F (2009). Personalized copy number and segmental duplication maps using next-generation sequencing.. Nature Genetics.

[14] Hormozdiari F, Hajirasouliha I, Dao P, Hach F, Yorukoglu D (2010). Next-generation VariationHunter: combinatorial algorithms for transposon insertion discovery.. Bioinformatics.

[15] Xie C, Tammi MT (2009). CNV-seq, a new method to detect copy number variation using high-throughput sequencing.. Bmc Bioinformatics.

[16] Durbin RM, Altshuler DL, Abecasis GR, Bentley DR, Chakravarti A (2010). A map of human genome variation from population-scale sequencing.. Nature.

[17] Huang J, Gusnanto A, O'Sullivan K, Staaf J, Borg A (2007). Robust smooth segmentation approach for array CGH data analysis.. Bioinformatics.

[18] Ben-Yaacov E, Eldar YC (2008). A fast and flexible method for the segmentation of aCGH data.. Bioinformatics.

[19] Pique-Regi R, Monso-Varona J, Ortega A, Seeger RC, Triche TJ (2008). Sparse representation and Bayesian detection of genome copy number alterations from microarray data.. Bioinformatics.

[20] Redon R, Ishikawa S, Fitch KR, Feuk L, Perry GH (2006). Global variation in copy number in the human genome.. Nature.

[21] LaFramboise T, Winckler W, Thomas RK (2009). A flexible rank-based framework for detecting copy number aberrations from array data.. Bioinformatics.

[22] Carter NP (2007). Methods and strategies for analyzing copy number variation using DNA microarrays.. Nat Genet.

[23] Park H, Kim JI, Ju YS, Gokcumen O, Mills RE (2010). Discovery of common Asian copy number variants using integrated high-resolution array CGH and massively parallel DNA sequencing.. Nature Genetics.

[24] Conrad DF, Pinto D, Redon R, Feuk L, Gokcumen O (2010). Origins and functional impact of copy number variation in the human genome.. Nature.

[25] Pique-Regi R, Ortega A, Asgharzadeh S (2009). Joint estimation of copy number variation and reference intensities on multiple DNA arrays using GADA.. Bioinformatics.

[26] Rueda OM, Diaz-Uriarte R (2009). Detection of recurrent copy number alterations in the genome: taking among-subject heterogeneity seriously.. Bmc Bioinformatics.

[27] Zhang NR, Siegmund DO, Ji HL, Li JZ (2010). Detecting simultaneous changepoints in multiple sequences.. Biometrika.

[28] Zhang QY, Ding L, Larson DE, Koboldt DC, McLellan MD (2010). CMDS: a population-based method for identifying recurrent DNA copy number aberrations in cancer from high-resolution data.. Bioinformatics.

[29] Shah SP (2008). Computational methods for identification of recurrent copy number alteration patterns by array CGH.. Cytogenetic and Genome Research.

[30] Bleakley K, Vert J-P (2009). Joint segmentation of many aCGH profiles using fast group LARS.. HAL - CCSD.

[31] Diskin SJ, Eck T, Greshock J, Mosse YP, Naylor T (2006). STAC: A method for testing the significance of DNA copy number aberrations across multiple array-CGH experiments.. Genome Research.

[32] Willenbrock H, Fridlyand J (2005). A comparison study: applying segmentation to array CGH data for downstream analyses.. Bioinformatics.

[33] Lai WR, Johnson MD, Kucherlapati R, Park PJ (2005). Comparative analysis of algorithms for identifying amplifications and deletions in array CGH data.. Bioinformatics.

[34] Yu TW, Ye H, Sun W, Li KC, Chen ZG (2007). A forward-backward fragment assembling algorithm for the identification of genomic amplification and deletion breakpoints using high-density single nucleotide polymorphism (SNP) array.. Bmc Bioinformatics.

[35] Tibshirani R, Wang P (2008). Spatial smoothing and hot spot detection for CGH data using the fused lasso.. Biostatistics.

[36] Picard F, Robin S, Lavielle M, Vaisse C, Daudin JJ (2005). A statistical approach for array CGH data analysis.. Bmc Bioinformatics.

[37] Eilers PHC, de Menezes RX (2005). Quantile smoothing of array CGH data.. Bioinformatics.

[38] Hupe P, Stransky N, Thiery JP, Radvanyi F, Barillot E (2004). Analysis of array CGH data: from signal ratio to gain and loss of DNA regions.. Bioinformatics.

[39] Marioni JC, Thorne NP, Tavare S (2006). BioHMM: a heterogeneous hidden Markov model for segmenting array CGH data.. Bioinformatics.

[40] Kim JI, Ju YS, Park H, Kim S, Lee S (2009). A highly annotated whole-genome sequence of a Korean individual.. Nature.

