# Supplementary material for: A Multi-Sample Based Method for Identifying Common CNVs in Normal Human Genomic Structure Using High-Resolution aCGH Data
Source: PLoS One. 2011 Oct 31;6(10):e26975. doi: 10.1371/journal.pone.0026975 (PMC3205051; doi:10.1371/journal.pone.0026975)
Supplement: File S1 — Five supplemental tables. (DOC) [file pone.0026975.s005.doc]

**SUPPORTING INFORMATION**

**A Multi-sample based Method for Identifying Common CNVs in Normal Human Genomic Structure using High-resolution aCGH Data**

Chihyun Park^1^, Jaegyoon Ahn ^1^, Youngmi Yoon^2^, Sanghyun Park^1§^

^1^Department of Computer Science, Yonsei University, Seoul, South Korea

^2^Division of Information Engineering, Gachon University of Medicine and Science, Incheon, South Korea

^§^Corresponding author

## Table 1: The optimal parameter set and its f1 score for each chromosome using 40 High-resolution aCGH Data

| Chromosome | Optimal *θ_CNVZ_* and *θ_dist_* | Optimal *θ_CNVZ_* and *θ_outlier_* | F1 score | Quantity of  findings | Ratio of findings |
| --- | --- | --- | --- | --- | --- |
| chr1 | *θ_CNVZ_*= 0.4 *θ_dist_* =0.085 | *θ_PCC_*= 0.8 *θ_outlier_* =3.5 | 0.626 | 15,301,400 Bp | 6.19 |
| chr2 | *θ_CNVZ_*=0.5 *θ_dist_* =0.080 | *θ_PCC_*= 0.8 *θ_outlier_* =3.5 | 0.577 | 7,705,910 Bp | 3.17 |
| chr3 | *θ_CNVZ_*=0.6 *θ_dist_* =0.115 | *θ_PCC_*= 0.8 *θ_outlier_* =3.5 | 0.513 | 3,561,800 Bp | 1.79 |
| chr4 | *θ_CNVZ_*=0.6 *θ_dist_* =0.060 | *θ_PCC_*= 0.8 *θ_outlier_* =3.5 | 0.418 | 3,607,640 Bp | 1.89 |
| chr5 | *θ_CNVZ_*=0.4 *θ_dist_* =0.055 | *θ_PCC_*=0.75 *θ_outlier_* =3.5 | 0.586 | 6,715,320 Bp | 3.71 |
| chr6 | *θ_CNVZ_*=0.4 *θ_dist_* =0.100 | *θ_PCC_*= 0.8 *θ_outlier_* =3.5 | 0.608 | 6,654,250 Bp | 3.89 |
| chr7 | *θ_CNVZ_*=0.4 *θ_dist_* =0.080 | *θ_PCC_*= 0.8 *θ_outlier_* =3.5 | 0.436 | 7,785,270 Bp | 4.90 |
| chr8 | *θ_CNVZ_*=0.4 *θ_dist_* =0.130 | *θ_PCC_*= 0.8 *θ_outlier_* =3.5 | 0.347 | 5,473,930 Bp | 3.78 |
| chr9 | *θ_CNVZ_*= 0.4 *θ_dist_* =0.065 | *θ_PCC_*= 0.8 *θ_outlier_* =3.5 | 0.467 | 7,109,280 Bp | 5.07 |
| chr10 | *θ_CNVZ_*=0.6 *θ_dist_* =0.040 | *θ_PCC_*= 0.8 *θ_outlier_* =3.5 | 0.571 | 6,179,239 Bp | 4.57 |
| chr11 | *θ_CNVZ_*=0.6 *θ_dist_* =0.100 | *θ_PCC_*= 0.8 *θ_outlier_* =3.5 | 0.451 | 1,967,360 Bp | 1.46 |
| chr12 | *θ_CNVZ_*=0.4 *θ_dist_* =0.095 | *θ_PCC_*= 0.8 *θ_outlier_* =3.5 | 0.588 | 3,343,710 Bp | 4.60 |
| chr13 | *θ_CNVZ_*=0.5 *θ_dist_* =0.100 | *θ_PCC_*= 0.8 *θ_outlier_* =3.5 | 0.330 | 1,213,490 Bp | 1.26 |
| chr14 | *θ_CNVZ_*=0.3 *θ_dist_* =0.075 | *θ_PCC_*= 0.8 *θ_outlier_* =3.5 | 0.594 | 7,553,540 Bp | 8.56 |
| chr15 | *θ_CNVZ_*=0.4 *θ_dist_* =0.100 | *θ_PCC_*= 0.8 *θ_outlier_* =3.5 | 0.666 | 6,760,880 Bp | 8.24 |
| chr16 | *θ_CNVZ_*=0.4 *θ_dist_* =0.060 | *θ_PCC_*= 0.8 *θ_outlier_* =3.5 | 0.584 | 6,589,790 Bp | 7.42 |
| chr17 | *θ_CNVZ_*=0.4 *θ_dist_* =0.085 | *θ_PCC_*= 0.8 *θ_outlier_* =3.5 | 0.560 | 5,111,120 Bp | 6.50 |
| chr18 | *θ_CNVZ_*=0.5 *θ_dist_* =0.085 | *θ_PCC_*= 0.8 *θ_outlier_* =3.5 | 0.537 | 1,735,730 Bp | 2.28 |
| chr19 | *θ_CNVZ_*=0.5 *θ_dist_* =0.090 | *θ_PCC_*= 0.8 *θ_outlier_* =3.5 | 0.679 | 3,030,550 Bp | 4.75 |
| chr20 | *θ_CNVZ_*=0.5 *θ_dist_* =0.095 | *θ_PCC_*= 0.8 *θ_outlier_* =3.5 | 0.345 | 1,097,320 Bp | 1.76 |
| chr21 | *θ_CNVZ_*=0.5 *θ_dist_* =0.100 | *θ_PCC_*= 0.8 *θ_outlier_* =3.5 | 0.458 | 971,633 Bp | 2.61 |
| chr22 | *θ_CNVZ_*=0.5 *θ_dist_* =0.075 | *θ_PCC_*= 0.8 *θ_outlier_* =3.5 | 0.787 | 3,476,988 Bp | 9.88 |
| chr23 | *θ_CNVZ_*=0.6 *θ_dist_* =0.045 | *θ_PCC_*= 0.8 *θ_outlier_* =3.5 | 0.510 | 4,298,820 Bp | 2.78 |

## Table 2: Smoothing algorithm of MGVD

| 1 | **set** all *r*_i,j_ from *N X M* |
| --- | --- |
| 2 | **for** each sample *N* |
| 3 | **set** *i* to current sample |
| 4 | **for** each position *M* |
| 5 | **set** *j* to current position |
| 6 | **set** *win_i_*,*_j_* to *w*-sized window for moving average |
| 7 | **if** *j* ≥ *w*/2 and *j* + *w*/2 ≤ *M* **then** |
| 8 | assign elements from *r*_i,(j-w/2)_ to *r*_i,(j+w/2)_ to *win_i_*,*_j_* |
| 9 | **else if** *j < w*/2 **then** |
| 10 | assign elements from *r*_i, 0_ to *r*_i,(j+w/2)_ to *win_i_*,*_j_* |
| 11 | **else** |
| 12 | assign elements from *r*_i,( j-w/2)_ to *r*_i_,*_M_* to *win_i_*,*_j_* |
| 13 | **end if** |
| 14 | *s_i,j_* ← average of *win_i_*,*_j_* |
| 15 | **end for** |
| 16 | **end for** |

## Table 3: Segmentation algorithm of MGVD

| 1 | **set** *SEG* is set of *SEG_i,j_* |
| --- | --- |
| 2 | **set** *BP* is set of break points |
| 3 | **for** each position *M* |
| 4 | **set *i*** to current position |
| 5 | **set** *N X 1* sub-matrix *V*_i_ using *s_N_*,*_i_* |
| 6 | **set** *N X 1* sub-matrix *V*_i+1_ using *s_N_*,*_i_*_+1_ |
| 7 | **if** *PCC*(*V*_i_ , *V*_i+1_) < *θ_pcc_* or *MD*(*V*_i_ , *V*_i+1_) ≥ *θ_dist_* **then** |
| 8 | **store** *i* to *BP* |
| 9 | **end if** |
| 10 | **end for** |
| 11 | **for** each element of *BP* |
| 12 | **set** *b* to current break point(position) |
| 13 | **if** *b* is the first element of *BP* **then** |
| 14 | *SEG*_i,j_ ← *SEG*_0_,*_b_* |
| 15 | **else** if *b* is the last element of *BP* **then** |
| 16 | *SEG*_i,j_ ← *SEG_b_*_+1_,**_M_** |
| 17 | **else** |
| 18 | **set** *b** is the previous element of *b* |
| 19 | *SEG*_i,j_ ← *SEG_b_*_*+1_,***_b_*** |
| 20 | **end if** |
| 21 | **end for** |

## Table 4: Clustering algorithm of MGVD

| 1 | **for** each *SEG* |
| --- | --- |
| 2 | **set** clustering objects *O* from *SEG* |
| 3 | **set** *C* with initial *k* points as *k*-centroids |
| 4 | compute distance among ∀*c*∈*C* and ∀*o*∈*O* |
| 5 | assign ∀*o*∈*O* to its closest *c* and make *CLU* |
| 6 | **for** each *clu*, ∀*clu*∈*CLU* |
| 7 | recompute each centroid with current cluster memberships |
| 8 | **set** *C_new_* to set of new centroids |
| 9 | **end for** |
| 10 | **if** *C_new_* ≠ *C* **then** |
| 11 | **goto** line 5 and replace *C* to *C_new_* |
| 12 | **else** |
| 13 | complete clustering |
| 14 | **return** *CLU_i_* = set of final clusters in *i_th_* segment |
| 15 | **end if** |
| 16 | **end for** |

## Table 5: Determination algorithm of CNVZ and CNV

| 1 | **set** *CNVZ^*^* to empty set |
| --- | --- |
| 2 | **set** *CNVZ* to empty set |
| 3 | **set** *CNVs_sample_* to empty set |
| 4 | **for** each *CLU* |
| 5 | *CLU* is set of *k* clusters |
| 6 | score **←** Compute *f(CLU_i_)* |
| 7 | **if** *θ_outlier_* > score > *θ_cnvz_* **then** |
| 8 | **store** *CLU* to *CZ*_i,j_ |
| 9 | **end if** |
| 10 | **end for** |
| 11 | *CNVZ** **←** all *CZ*_i,j_ |
| 12 | **for** each sample |
| 13 | identify *CNVs* of sample from *CNVZ^*^* |
| 14 | merging and pruning for identified *CNVs* |
| 15 | **store** all *CNVs* to *CNVs_sample_* |
| 16 | **end for** |
| 17 | **store** *CNVZ* ← merging and pruning *CNVZ^*^* |
